# Cross-cultural validation of the FACE-Q Satisfaction with Facial Appearance Overall Scale (FACE-Q SFAOS) in Brazilian rhytidoplasty patients

**DOI:** 10.6061/clinics/2020/e1568

**Published:** 2020-07-27

**Authors:** José Teixeira Gama, Luís Antônio Rossetto, Nathalya Botelho Brito, Daniela Francescato Veiga, Lydia Masako Ferreira

**Affiliations:** Programa de Pos-graduacao em Cirurgia Translacional Universidade Federal de Sao Paulo, Sao Paulo, SP, BR

**Keywords:** Validation Studies, Surveys and Questionnaires, Rhytidoplasty, Plastic Surgery

## Abstract

**OBJECTIVES:**

This study aimed to culturally validate the FACE-Q - Satisfaction with Facial Appearance Overall Scale (Face-Q SFAOS) in a population of Brazilian rhytidoplasty patients.

**METHOD:**

Authorization for the translation and validation of the questionnaire was obtained from the FACE-Q SFAOS distribution rights holders. The FACE-Q SFAOS was translated and then back-translated. For cultural validation, a total of 57 women were selected 5 to 8 months after undergoing rhytidoplasty. Twenty of them participated in the cultural adaptation, 30 participated in the reproducibility analysis, and 57 participated in the construct validation.

**RESULTS:**

The analysis identified two factors (general appearance and face geometry) that exhibited excellent internal consistency. The total satisfaction score, which comprised nine items, also presented excellent internal consistency. Good reproducibility was found for Overall Appearance, Geometry and Total. There was a difference in the satisfaction means (total and factors) between procedure locations; patients undergoing frontal, upper eyelid and lower eyelid procedures were less satisfied than those who did not undergo such procedures. Satisfaction was higher with geometry than with overall face appearance.

**CONCLUSION:**

The FACE-Q SFAOS was adapted to the cultural context of Brazilian rhytidoplasty patients and was reproducible, and the scale exhibited face, content and construct validity.

## INTRODUCTION

Interest in facial rejuvenation has been growing, resulting in an increase in demand for plastic surgery, especially rhytidoplasty (1-7). In the USA, there was a 21.9% increase in the number of rhytidoplasty procedures from 2016 to 2017 (8). In 2017, according to the International Society of Aesthetic Plastic Surgery (ISAPS), Brazil ranked first in procedures performed on the face in general and fourth in considering other facial rejuvenation procedures (9). According to the American Society of Plastic Surgeons (ASPS), there was a 2% increase in minimally invasive facial aesthetic procedures from 2016 to 2017 (10).

Satisfaction with facial appearance is undoubtedly the most important outcome for facial rejuvenation patients (4). However, research assessing outcomes from the patient's perspective is still limited (6,11). This kind of assessment is crucial for making the best clinical decision, the informed consent process, managing postoperative expectations, assessing treatment satisfaction and efficacy, and determining the cost-utility of interventions (3,7,11,12).

In recent decades, aging has begun to increasingly take on a negative connotation. Intrinsic aspects of aging are often considered medical and social problems that need to be addressed by health professionals (1,2). Another current phenomenon is the emergence of a large number of individuals with concerns about aging at a very early age (13). This tendency of younger individuals to seek treatments for skin rejuvenation emphasizes the importance of including instruments that measure patient-reported outcomes (PROs) in clinical practice, as some individuals may have unrealistic expectations of what they are looking for and what the treatment itself may offer (2,3,13,14).

To address the shortage of PRO instruments available to plastic surgeons and dermatologists, in 2010, Klassen et al. developed the FACE-Q, a set of scales for use in patients undergoing aesthetic and surgical facial procedures that can be used independently by clinicians and researchers (6,15). The FACE-Q, unlike other PRO questionnaires, is comprehensive (16). In addition to capturing changes in patient satisfaction with overall facial appearance due to aesthetic intervention, it assesses the satisfaction of specific facial regions and patients' perception of appearance in relation to their age. The breadth of this tool can eliminate the need to combine multiple instruments. In this context, and according to the authors of FACE-Q, a key patient outcome scale is Satisfaction with Facial Appearance Overall (6,15).

Recently, Bustillo and colleagues published the Brazilian version of the complete FACE-Q questionnaire (17), which they tested in patients who had undergone any surgical or nonsurgical facial cosmetic procedure. The current study provides cross-cultural validation of the FACE-Q Satisfaction with Facial Appearance Overall Scale for a significant sample of Brazilian rhytidoplasty patients.

## METHODS

The study was approved by the Research Ethics Committee of the Federal University of São Paulo (UNIFESP). The precepts contained in the Declaration of Helsinki were strictly followed, and all participants signed an informed consent form.

Fifty-eight women, aged 40 to 75 years old, were enrolled between five and eight months after undergoing rhytidoplasty. Women were consecutively selected at the Plastic Surgery Outpatient Clinics of the São Paulo Hospital of UNIFESP, the Ipiranga Hospital and the General Hospital Vila Penteado in São Paulo. Sampling was non-probabilistic. Patients with a history of untreated psychiatric illness, with an established diagnosis of body dysmorphic disorder, or who underwent any other facial rejuvenation procedures such as peeling, fillings, or botulinum toxin within the previous year were not included. Patients who withdrew their consent at any stage of the study were excluded.

The present study was conducted after the Mapi Research Trust, holder of the scale distribution rights, granted us permission to translate, culturally adapt and validate the instrument into Brazilian Portuguese and after the translation and back-translation step.

The total sample size and subsamples for the cultural adaptation, reproducibility and validity phases were calculated according to the methodology described by Guillemin et al. (18-20) and Gandek and Ware (21).

### The instrument

The FACE-Q - Satisfaction with Facial Appearance Overall Scale consists of ten items that assess the current perception of facial appearance for symmetry, harmony, proportion, freshness or vitality, its appearance over time (such as rested facial appearance), its appearance at the end of day, its appearance when waking up, its appearance in the face of the brightest light, and the picture of oneself and one's profile (side view or contour). Therefore, the scale assesses satisfaction with facial appearance in the presence of some scenarios (6). It was developed by Klassen et al. in 2010 (15), and in 2013, Pusic et al. (6) evaluated the psychometric properties of the instrument (6,15).

The items have four response options (very dissatisfied, somewhat dissatisfied, somewhat satisfied, and very satisfied), which generate scores ranging from 10 to 40 and are summed to a total score ranging from 0 to 100 (6,15). Higher scores indicate greater satisfaction or better quality of life.

### Translation

The original version of the FACE-Q - Satisfaction with Facial Appearance Overall Scale was translated from English to Brazilian Portuguese by two independent translators. Only one of the translators was informed about the objectives of the study; this was done to achieve a conceptual rather than strictly literal translation. Both translations were reviewed by a multidisciplinary committee composed of four plastic surgeons, one nurse and one psychologist, all with PhD degrees. The multidisciplinary group analyzed all the questions to find possible mistakes made during the translation phase and to assess the applicability of each question.

After the group evaluation, a version of the scale in Portuguese was produced based on elements from both previously translated versions (18). The following equivalences were considered when translating the scale: idiomatic, semantic, conceptual and cultural.

The Portuguese version was then translated into English (back-translation) by two separate independent translators who were unaware of the original scale or purpose of the study. Both translated versions of the scale were analyzed and compared with the original by the same multidisciplinary group to correct possible errors or discrepancies in the translation process (19). This English version was approved by the author of the original instrument. From this analysis and approval came the final version, in Brazilian Portuguese, which was duly adapted to the linguistic and cultural context of the population while maintaining all the essential characteristics of the original scale in English (20).

### Cultural adaptation or pretest

At this stage, the FACE-Q Satisfaction with Facial Appearance Overall Scale was applied to 20 patients. Patients were asked to explain each question in their own words and to suggest changes in its formulation (adaptation of the question). The interviews were conducted face to face.

All patients understood that the items on the scale were related to concerns and levels of satisfaction with their facial appearance, thus indicating the scale’s face validity at this stage (analyzing whether the instrument measures what it proposes to measure, or whether the items offered no resistance) and content validity (defined as the degree to which each item is relevant in measuring the content of the target population) (21). The final version was obtained when patients had no further doubts and the multidisciplinary team reached a consensus (20).

### Evaluation of psychometric properties

After translation and cultural adaptation, based on the model recommended by Beaton et al. in 2000 (20), we proceeded to evaluate the scale’s psychometric properties, as suggested by Gandek and Ware (21). The reliability (internal consistency by scale and factors and intraobserver reproducibility by means of factors assessed in different periods) was tested. The construct validity by divergent validity (by analyzing the differences between the satisfaction dimensions by patient characteristics) and by factor analysis (latent construct dimensionality analysis) were also tested.

In this study, we investigated the initial scale dimensionality of 10 items (6,15) to verify whether the concept of satisfaction was one-dimensional (via factor analysis), which defined the outline of the psychometric analysis of the FACE-Q - Satisfaction with Facial Appearance Overall Scale. Reliability was tested on 57 patients by Cronbach's alpha, a measure of internal consistency.

The reproducibility of the FACE-Q SFAOS was tested by two interviews with 30 patients submitted to the instrument application by the same interviewer (E1+E1) with an interval of seven days between applications. This instrument is self-applicable; however, the researcher was present during the application to clarify the possible doubts. This phase is used to verify the accuracy of the instrument in measuring the properties for which it was designed (19,20).

For construct validity, factor analyses were used and evaluated the scale's dimensionality, that is, its latent construct, and the divergent validity was tested considering total satisfaction and satisfaction factors by characteristics of 58 patients.

### Statistical analysis

Cronbach's alpha was used to assess internal consistency. For reproducibility, Pearson's correlation coefficients (r) and intraclass correlations (ICC) were used.

The dimensionality of the 10-item scale was assessed by factor analysis using the principal component method and VARIMAX orthogonal rotation. The criterion for selecting the number of factors was eigenvalues above value one. Factorial analysis allows the variance of each item to be decomposed into two parts: common variance and specific variance. The portion of the common variance - due to common factors - is called commonality. The exclusion criterion for items was having a commonality less than 0.6, because these items were poorly represented in the factor analysis.

The Kaiser-Meyer-Olkin (KMO) sample adequacy coefficient and the Bartlett sphericity test assessed the overall significance of all correlations between items. The factors were generated as sum and then rescaled to vary from 0 to 100, being compared by patient characteristics via Student's t-test (for comparison of two means) or analysis of variance (ANOVA; for comparison of more than two averages).

For normality in data distribution and homoscedasticity, the Kolmogorov-Smirnov test and Levene test, respectively, were used. When the homoscedasticity assumption was violated, the degrees of freedom of the F statistics were corrected using the Brown-Forsythe correction. When the normality assumption was violated, the means were compared using the nonparametric Mann-Whitney test (for comparison of two averages) or Kruskal-Wallis (for comparison of more than two averages). Once the mean differences were detected in the Kruskal-Wallis test, the differences were localized via the Dunn-Bonferroni test, maintaining a global significance level of 5%.

The levels of face satisfaction were compared using Student’s t test for paired samples. Linear associations between the dimensions of face satisfaction and patient characteristics in numerical form were evaluated via Pearson correlation.

Statistical Package for Social Sciences (SPSS) 20.0 (SPSS Inc., Chicago, IL, USA) and Stata 12 (StatCorp, College Station, Texas, USA) were used for data analysis. For all statistical tests, *p*<0.05 was considered statistically significant.

## RESULTS

The Brazilian version of the FACE-Q SFAOS was administered to 58 patients. One of the patients was excluded because she withdrew her consent, resulting in a final sample of 57 patients.

The patients had no doubts about the items and considered the instrument easy to understand. The average response time to the instrument was approximately five minutes.

Most women were menopausal (77.6%) and lived in a stable union (60.3%). Age ranged from 44 to 74 years (mean 58.3; standard deviation 6.3 years), and 51.1% completed high school. It was also noted that 51.7% were using other medications, and more than 30% of the patients underwent partial rhytidoplasty (interventions limited to the frontal, middle third of the face or upper eyelid). On average, the patients had undergone 1.7 procedures (SD=1.6).

The initial factor analysis of ten items pointed to the existence of two factors that explained 69.4% of the total variance of the items. In the subsequent factor analysis, item “d. With the appearance of your face at the end of the day”, had a commonality of 0.497, which was less than 0.6.

After excluding item d, the factor analysis resulted in two factors that explained 73.1% of the total variance of the remaining nine items. From the results presented in Table 1, the two factors can be interpreted as follows:

**Factor 1. Overall Face Appearance -** High values in this factor indicate greater satisfaction with respect to the following five items: “j. With the appearance of your face in bright (or bright) light”, “f. With the rested look of your face”, “e. With the freshness of your face”, “i. How your face looks when you wake up” and “h. How your face looks in photos”.

**Factor 2. Face Geometry -** High values in this factor indicate greater satisfaction with respect to the following four items: “a. With the symmetry of your face”, “b. With the harmony of your face”, “g. With the appearance of your profile (side view)” and “c. With the proportion of your face”.

It can be noted that the internal consistencies, assessed by Cronbach's alpha (α), for both factor 1 (α=0.876) and factor 2 (α=0.903) were very good (Table 1). The total satisfaction score comprising all nine items also showed excellent internal consistency (α=0.920).

Good reproducibility was observed for facial satisfaction scores: Overall Appearance (0.983), Geometry (0.970) and Total (0.989) (Table 2). The dimensions of facial satisfaction by characteristics allowed us to analyze the difference between the parts, that is, the divergent validity of the scale.

No mean differences in total satisfaction scores and dimensions (General Appearance and Face Geometry) were identified based on menopause, marital status, education, use of medications, or procedure location in the mid and cervical regions. However, as shown in Table 3, the patients who underwent intervention in the frontal region had significantly lower mean total satisfaction and dimensions (Overall Appearance and Face Geometry) than women who did not undergo procedures in this area.

Likewise, it was found that patients who underwent interventions on the upper eyelid had lower mean satisfaction (total and dimensions) than women who did not undergo procedures in this location (Table 4). Table 5 shows that patients who underwent interventions in the lower eyelid presented lower average satisfaction (*p*=0.014) and overall appearance (*p*=0.021) than women who did not. For the geometric aspect, there were no differences in means (*p*=0.055) (Table 5).

Weak but significant negative correlations were found between age and total score (r=-0.293; *p*=0.027) and between age and geometry (r=-0.328; *p*=0.013), indicating that older patients had lower total satisfaction and less satisfaction with the facial geometric aspect. In addition, weak but significant negative correlations were also observed between the number of procedures and total score (r=-0.300; *p*=0.023) and between the number of procedures and overall appearance (r=-0.289; *p*=0.028), indicating that the higher the number of interventions is, the lower the total satisfaction and satisfaction with respect to the general appearance of the face.

Means of factors or dimensions differed between facial satisfaction aspects (*p*<0.001). In general, patients were more satisfied with the geometry factor than with the overall face appearance factor.

After rescheduling, the total satisfaction, general appearance and geometry scores were rounded to be presented as integers (to facilitate the operationalization of their use). The intraclass correlations for the three scores (0 to 100 in the original and rounded forms) were 1.00 (*p*<0.001), indicating that they are equivalent.

## DISCUSSION

The FACE-Q - Satisfaction with Facial Appearance Overall Scale was culturally adapted and tested for reproducibility and construct validity in rhytidoplasty patients. This is a short, objective and easy-to-answer scale that captures information specific to satisfaction with overall appearance and facial geometry and has been used in many studies (5,7,11,16). The general guidelines for cross-cultural adaptation of instruments were carefully followed to ensure the quality of the Brazilian version of the instrument.

The word “rosto” was determined, in the cultural adaptation phase, to mark the face, since Brazilian patients understood this word better. One item on the scale was excluded, and only 50% explained satisfaction; the rest were related to the individual's particularity. This modification was statistically possible because it is an instrument with only ten items.

The total scores (nine items) and factors of the Brazilian version for the conversion of the four-point Likert scale were rounded to be presented as an integer to facilitate the operation of its use. The intraclass correlations for the three scores (0 to 100 in the original and rounded forms) were 1.000 (*p*<0.001), indicating that they are equivalent.

The overall value of Cronbach's alpha (α) found in the present study for the nine-item scale was 0.920, and for both factors or dimensions, it was 0.876 and 0.903, which indicate excellent reliability (22). The original ten-item scale had a reliability of α=0.950 (6). The Pearson's correlations between factors, evaluated at different times, showed that the effect size was very high, which means that the sample was adequate. Pusic et al. (6) reported moderate effect sizes in their study by assessing patients' satisfaction and response percentage after the facial procedure (6).

The latent construct “satisfaction” revealed not to be one-dimensional, bringing new data to the FACE-Q Satisfaction with Facial Appearance Overall Scale. This phenomenon can be explored in further studies using the validated questionnaire as a basis.

The patients scored high values for the total satisfaction score (79.2; SD=20.5) and for the two dimensions of overall face appearance (74.9; SD=22.1) and face geometry (84,6; SD=21.9). These data indicate satisfaction with the result of the surgical treatment. These results are similar to those found in the literature for this type of aesthetic procedure (7,12,16), which demonstrates the effectiveness of the questionnaire regarding patient satisfaction. These satisfaction scores (total and dimensions General face appearance and face geometry) measured by the scale were evaluated by characteristics that showed differences in the location of the frontal and upper and lower eyelid procedures. That is, the scale was able to distinguish between satisfied and unmet patients considering specific aesthetic and facial features.

Satisfaction was found to be associated with the procedure site. Patients who underwent interventions in the frontal and eyelid regions were less satisfied with the overall appearance of the face and face geometry when compared to those who did not undergo this type of surgical procedure, a result different from that found by Klassen et al. (24). In fact, facial procedures may cause transient identity changes that are expressed as dissatisfaction with the results (25,26). Procedures in the frontal and upper eyelid regions may alter facial expression and interfere with the satisfaction result, as observed in this study. This was also noted when it was found that procedures at the lower eyelid region did not change satisfaction with facial geometry, perhaps because the physical change was not so pronounced. However, these differences in perceived outcome may differ between patients and surgeons, revealing the complexity of satisfaction after rhytidoplasty and other facial aesthetic procedures (3,7,14).

Recently, Bustillo et al. published the Brazilian version of the entire FACE-Q Questionnaire (17). Their study is relevant and has merit, as it involved the translation and linguistic validation of an instrument composed of 353 questions. However, the cross-cultural adaptation may have been biased, since only 7 patients were enrolled, of a wide age range, without gender specification, submitted to surgical or non-surgical facial cosmetic procedures (17). The present study complements the study of Bustillo et al. by providing further cross-cultural validation of the FACE-Q Satisfaction with Facial Appearance Overall Scale, performed in a significantly larger sample (n=57) of rhytidoplasty patients selected with very strict eligibility criteria.

In this study, only women were studied. Women are more influenced by sociocultural patterns, and studies have reported the high prevalence of females undergoing this type of aesthetic procedure (1,2,5,6,15,23,24).

The postoperative evaluation time of five to eight months after rhytidoplasty was short but similar to that reported by Alves et al. (1), who assessed outcomes after two and six months, and by Pusic et al. (6), who evaluated patients six months after rhytidoplasty (1,6). In this line of thought, Klassen et al. (24) stated that there were few studies that assessed patients pre- and postoperatively; thus, prospective and longitudinal studies are still needed in this area (24).

Satisfaction with appearance and improved quality of life are the most important results for patients undergoing facial aesthetic procedures (4,11,14,15). Nevertheless, although there is an increased interest for these procedures (3,7,24,26,27), the use of reliable and validated instruments is still scarce (25,28). Therefore, patient perspective-based (PRO) instruments are considered to be important and state-of-the-art tools in regard to evidence-based outcomes (11,27,28).

## CONCLUSION

The FACE-Q - Satisfaction with Facial Appearance Overall Scale was adapted to the cultural context of Brazilian rhytidoplasty patients; it was reproducible, and it exhibited face, content and construct validity.

## AUTHOR CONTRIBUTIONS

Gama JT was responsible for the conception of the study, data collection, analysis and interpretation, and manuscript writing. Rossetto LA was responsible for the conception and design of the study, and critical review of the manuscript for important intellectual content. Brito NB was responsible for the manuscript editing and review for important intellectual content. Veiga DF was responsible for the conception and design of the study, and manuscript critical review for important intellectual content. Ferreira LM was responsible for the conception and design of the study, and critical review of the manuscript for important intellectual content. All of the authors, except Gama JT read and approved the final version of the manuscript.

## Figures and Tables

**Table 1:** Factorial loads, eigenvalues, percentage of explained variance and Cronbach’s alpha coefficient of the two factors of the Face-Q SFAOS.

Factors			
Face appearance	1	2	Commonality
j. In light	0.833	0.355	0.820
f. Rest	0.778	0.239	0.663
e. Cool	0.749	0.309	0.656
i. At wake up	0.749	0.239	0.617
h. In photos	0.625	0.476	0.617
a. Symmetry	0.204	0.943	0.930
b. Balance	0.331	0.887	0.896
g. Side view	0.419	0.758	0.750
c. Proportion	0.433	0.665	0.630
Eigenvalues	3.32	3.25	
Percentage (%) of total variance explained	36.94	36.16	
Cumulative Percentage (%) of Total Variance Explained	36.94	73.10	
Cronbach’s Alpha	0.876	0.903	

Varimax Rotation.

KMO=0.832.

Bartlett-Chi Sphericity Test (36) = 386.56 (*p*<0.001).

N=57.

**Table 2:** Intraclass correlation and Pearson (r) correlation between factors evaluated at different times.

	Intraclass correlation		Pearson correlation		
	Estimate (CI95%)	*p*	Estimate (CI95%)	*p*	N
Total	0.989 (0.977; 0.995)	<0.001	0.991	<0.001	30
General appearance of the face	0.983 (0.965; 0.992)	<0.001	0.983	<0.001	30
Face geometry	0.970 (0.939; 0.986)	<0.001	0.980	<0.001	30

**Table 3:** Summary measures of facial satisfaction factors by procedure location - frontal region.

	Average	Standard deviation	Minimum	Maximum	1^st^ Quartile	Median	3^rd^ Quartile	N	*p*
**Total**									0.037^a^
Front	68.2	25.3	11.1	100.0	48.1	63.0	92.6	19	
Other regions	84.7	15.2	44.4	100.0	81.5	88.9	92.6	38	
**General appearance**									0.007
Front	64.2	25.1	13.3	100.0	46.7	60.0	86.7	19	
Other regions	80.7	18.7	20.0	100.0	73.3	86.7	93.3	38	
**Geometry**									0.020^a^
Front	73.2	27.6	8.3	100.0	50.0	75.0	100.0	19	
Other regions	90.4	16.0	33.3	100.0	83.3	100.0	100.0	38	

*p* - Descriptive level of Student's t test or Mann-Whitney (^a^).

**Table 4:** Summary measures of facial satisfaction factors by procedure location - upper eyelid.

	Average	Standard deviation	Min	Max	1^st^ Quartile	Median	3^rd^ Quartile	N	*p*
**Total**									0.002
Upper eyelid	70.7	19.0	40.7	100.0	53.7	74.1	88.9	22	
Other Regions	84.6	19.8	11.1	100.0	85.2	92.6	96.3	35	
**General appearance**									0.003
Upper eyelid	66.4	19.4	40.0	100.0	46.7	66.7	80.0	22	
Other Regions	80.7	22.3	13.3	100.0	73.3	86.7	98.3	35	
**Geometry**									0.003
Upper eyelid	76.1	22.0	33.3	100.0	56.3	83.3	100.0	22	
Other Regions	90.0	20.4	8.3	100.0	91.7	100.0	100.0	35	

*p* - Mann-Whitney test descriptive level.

**Table 5:** Summary measures of facial satisfaction factors by procedure location - lower eyelid.

	Average	Standard deviation	Min	Max	1^st^ Quartile	Med	3^rd^ Quartile	N	*p*
**Total**									0.014
Lower eyelid	71.8	19.6	40.7	92.6	48.1	77.8	88.9	16	
Other Regions	82.1	20.3	11.1	100.0	68.5	92.6	96.3	41	
**General appearance**									0.021
Lower eyelid	67.5	18.4	40.0	93.3	50.0	73.3	80.0	16	
Other Regions	78.3	23.0	13.3	100.0	66.7	86.7	95.0	41	
**Geometry**									0.055
Lower eyelid	77.1	23.5	33.3	100.0	52.1	83.3	100.0	16	
Other Regions	87.6	20.8	8.3	100.0	79.2	100.0	100.0	41	

*p* - Mann-Whitney test descriptive level.
