# Erratum for: Cross-cultural validation of the FACE-Q Satisfaction with Facial Appearance Overall Scale (FACE-Q SFAOS) in Brazilian rhytidoplasty patients

**DOI:** 10.6061/clinics/2020/e1568err

**Published:** 2020-10-05

## CLINICS 2020;75:e1568

In the article **Cross-cultural validation of the FACE-Q Satisfaction with Facial Appearance Overall Scale (FACE-Q SFAOS) in Brazilian rhytidoplasty patients**

Replace **Table 1** for:

**Table 1:** Factorial loads, eigenvalues, percentage of explained variance and Cronbach’s alpha coefficient of the two factors of the Face-Q SFAOS.

Factors			
Face appearance	1	2	Commonality
j. In light	0.833	0.355	0.820
f. Rest	0.778	0.239	0.663
e. Cool	0.749	0.309	0.656
i. At wake up	0.749	0.239	0.617
h. In photos	0.625	0.476	0.617
a. Symmetry	0.204	0.943	0.930
b. Balance	0.331	0.887	0.896
g. Side view	0.419	0.758	0.750
c. Proportion	0.433	0.665	0.630
Eigenvalues	3.32	3.25	
Percentage (%) of total variance explained	36.94	36.16	
Cumulative Percentage (%) of Total Variance Explained	36.94	73.10	
Cronbach’s Alpha	0.876	0.903	

Varimax Rotation.

KMO=0.832.

Bartlett-Chi Sphericity Test (36) = 386.56 (*p*<0.001).

N=57.

